# Cryoablation Allows the Ultimate De-escalation of Surgical Therapy for Select Breast Cancer Patients

**DOI:** 10.1245/s10434-023-14332-3

**Published:** 2023-09-28

**Authors:** Sonia Y. Khan, Jaclyn Cole, Zaina Habrawi, Michael W. Melkus, Rakhshanda Layeequr Rahman

**Affiliations:** Breast Center of Excellence and Department of Surgery, School of Medicine, Texas Tech University Health Sciences Center, Lubbock, TX USA

**Keywords:** Breast cancer, Cryoablation, Surgical resection, Surgical de-escalation

## Abstract

**Background:**

Widespread use of screening mammography has allowed breast cancer to be detected at earlier stages. This allows for increased customization of treatment and less aggressive management. De-escalation of therapy plays an important role in decreasing treatment burden and improving patient quality of life. This report examines cryoablation as the next step in the surgical de-escalation of breast cancer.

**Methods:**

Women with a diagnosis of clinically node-negative, estrogen receptor-positive (ER +), progesterone receptor-positive (PR +), human epidermal growth factor receptor 2-negative (HER2 −) infiltrating ductal carcinomas 1.5 cm or smaller underwent ultrasound-guided cryoablation. Either the Visica 2 treatment system (before 2020) or the ProSense treatment system (since 2020) was used to perform the cryoablation. Patients received mammograms and ultrasounds at a 6 months follow-up visit, and magnetic resonance images at baseline, then at 1 year follow-up intervals. Adjuvant therapy decisions and disease status were recorded.

**Results:**

This study enrolled 32 patients who underwent 33 cryoablation procedures (1 patient had bilateral cancer). One patient had a sentinel node biopsy in addition to clinical staging of the axilla. For all the patients, adjuvant endocrine therapy was recommended, and six patients (18.75%) received adjuvant radiation. Of the 32 patients, 20 (60.6%) have been followed up for 2 years or longer, with no residual or recurrent disease at the site of ablation.

**Conclusion:**

Cryoablation of the primary tumor foregoing sentinel node biopsy offers an oncologically safe and feasible minimally invasive office-based procedure option in lieu of surgery for patients with early-stage, low-risk breast cancer.

After Professor Bernard Fisher rejected the mechanistic model of breast cancer and proposed the revolutionary hypothesis of tumor biology at its inception that dictates its growth and spread, radical surgical resection of visible disease came into question.^1^ Whereas addressing primary disease remains pivotal in treatment of the breast based on newer understanding of tumor biology, the last five decades have witnessed remarkable de-escalation of surgery for breast cancer, from radical surgery to breast conservation, and from level 3 axillary dissections to sentinel node sampling.^2^ This can be attributed to the increase in screening and improvement in detection of breast cancer at an earlier stage^3^ as well as to the addition of radiation and systemic therapies, which open the possibilities for less aggressive surgical management.^1^

Totally non-invasive techniques such as high-intensity focused ultrasound (HIFU) and minimally invasive techniques such as cryoablation, radiofrequency ablation, laser ablation, and microwave ablation are being used in lieu of surgery.^4^ Cryoablation is emerging as a cost-effective^5^ ablative option because the procedure can be performed in an office setting under ultrasound guidance with excellent short-term oncologic, physical, sexual, and cosmetic outcomes.^5,6^ Cryoablation of low-risk small breast cancer in the ICE3 trial, a large multi-institutional study of 194 patients, showed a recurrence rate of a mere 2% in the short term.^7^

While minimally invasive techniques are being popularized to address the primary tumor, the need for axillary staging (traditionally achieved through a surgical procedure) is also being questioned because therapeutic decisions are based more on biology profiles of cancer in early disease.^8^ These paradigm shifts have led to a more tailored approach to the treatment of breast cancer, with particular emphasis on surgical de-escalation.^9,10^ This study documented the account of ultimate de-escalation of breast and axillary surgery for select patients using cryoablation.

## Methods

### Study Design

A single-institution longitudinal study was approved by the Institutional Review Board (TTUHSC IRB #L18-100) to offer cryoablation to patients age 50 years or older with early-stage, low-risk estrogen receptor-positive (ER +), progesterone receptor-positive (PR +), human epidermal growth factor receptor 2-negative (HER2 −) unifocal invasive ductal carcinoma 1.5 cm or smaller at its widest diameter without an extensive in situ component visible on ultrasound. The patients who underwent cryoablation had follow-up assessment with clinical exam, breast mammography, and ultrasound every 6 months as well as magnetic resonance imaging (MRI) at baseline, then every year for 2 years. Adjuvant chemotherapy and radiation were at the discretion of the medical oncologist and radiation oncologist, with the patient sharing in the decision-making.

### Cryoablation and Resection Techniques

Cryoablation was performed with local anesthesia in a clinical setting using either the Visica 2 treatment system (Sanarus Technologies, Inc., Pleasanton, CA, USA) or the ProSense treatment system (IceCure Medical, Caesarea, Israel) by a trained breast surgical oncologist. Ultrasound was used during the procedure to guide the probe to the appropriate location for proper tumor penetration and freezing as previously described in detail.^6^ Once the probe was visualized penetrating through the widest diameter of the tumor, freeze/thaw cycles were performed according to a tumor size-dependent predetermined algorithm adapted from ACOSOG Z-1072.^11^

### Data Collection and Statistical Analysis

Data were collected on tumor size, clinical stage, and adjuvant locoregional and systemic therapy. Follow-up patient information on disease status and compliance with recommended treatment was recorded at 6 month intervals. Patient status at the last follow-up appointment was recorded for this analysis. Data were summarized with descriptive statistics such as mean ± standard deviation, median (interquartile range), and count (percentage) depending on what was appropriate for the examined variable.

## Results

Between January 2017 and May 2023, 32 patients underwent 33 cryoablation procedures (1 patient had bilateral early-stage breast cancer, for which she received bilateral cryoablation). Over-the-counter acetaminophen and ibuprofen were used for post-procedure pain control.

All primary tumors were genomically profiled using 150-gene MammaPrint–BluePrint assays and confirmed to be luminal A subtype. Baseline patient and tumor characteristics are depicted in Table 1. The detailed therapeutic approach and outcomes are depicted in Table 2. Only one 51-year-old patient received sentinel node biopsy (SLNB) under general anesthesia at the request of the medical oncologist. Of the 32 patients, 6 (18.75%) received adjuvant radiation (3 [9.38%] with a whole-breast protocol and 3 [9.38%] with an accelerated partial-breast protocol).

**Table 1:** Patient and tumor characteristics

Characteristic *N*=32	Value *n* (%)
*Age (years)*	
Mean ± SD	71 ± 10.5
Median (range)	70 (50–91)
*Breast laterality (n = 33)*^a^	
Left	19 (57.6)
Right	14 (39.4)
*Cancer stage (n = 33)*^a^	
1A	9 (27.3)
1B	13 (39.4)
1C	11 (33.3)
*Tumor size by ultrasound (mm)*	
Mean ± SD	8.7 ± 3.5
Median (range)	8.0 (4.0–15)
*Follow-up (months)*	
≥ 24	20 (62.5)
12–24	8 (25.0)
6–12	2 (6.3)
< 6	3 (9.4)

*SD* standard deviation

^a^One patient had bilateral disease for which both lesions were treated with cryoablation

**Table 2:** Therapeutic approach and outcome

Age group (years)	No. of patients	SLNB *n* (%)	Adjuvant radiation *n* (%)	Adjuvant endocrine therapy *n* (%)	Status at last follow-up visit
50–60	4	1 (25)	3 (75)	4 (100)	All alive and disease-free.
61–70	14	0	3 (21.4)	14 (100)	All alive and disease-free.
71–80	10	0	0	9 (90)	One patient had regional disease at 18 months. She did not have SLNB and did not take endocrine therapy. She had delayed axillary dissection, started endocrine therapy, and remains disease-free after 5 years of follow-up evaluation. Of 10 patients, 9 are alive and disease-free at last follow-up.
≥ 81	4	0	0	4 (100)	All alive and disease free.

*SLNB* sentinel lymph node biopsy

By the end of May 2023, 20 patients (62.5%) had completed a 2-year follow-up period, and 12 patients (37.5%) had experienced more than 3 years of follow-up evaluation. One patient underwent axillary dissection for nodal disease diagnosed at the 18 months follow-up visit. She did not have SLNB and was not offered adjuvant radiation. Adjuvant endocrine therapy was recommended, but she reported not taking it at the time axillary disease was diagnosed. At this writing, she has been compliant with endocrine therapy after axillary dissection and remains disease-free at 5 years.

A second patient underwent excision for imaging suspicion of residual disease at 6 months, but the final pathology showed complete ablation of tumor. No major complications from the procedure were reported. One patient died 1 year after cryoablation due to unrelated causes.

## Discussion

In the late 1800s, antisepsis and anesthesia allowed surgeons to perform radical resection of breast cancers to affect cure.^12^ The Halstedian principle dictated that breast cancer spreads outward through invasion from original growth, thereby popularizing radical mastectomy until about 1975.^13^ This technique involved removing not only the breast, but also the chest muscles and axillary lymph nodes all in the same procedure to prevent local recurrence. It then was determined that a modified radical mastectomy could be performed, leaving the pectoralis muscles behind. On one hand, the paradigm shift proposed by Dr. Fisher that breast cancer is a systemic disease from the outset, challenged the doctrine of radical resection.^1^ On the other hand, the development of systemic therapies that achieved better survival further paved way to de-escalate surgical approaches to breast cancer.^14^ This conceptual change about the natural history of breast cancer coupled with the increase in screening mammograms finding breast cancers at earlier stages with smaller tumors but no nodal disease has opened a whole new opportunity for minimizing surgeries.

During the last five decades, tailored lumpectomies not only were determined to be feasible in terms of patient outcomes, but also were shown to offer a better quality of life than mastectomies.^15^ Although lumpectomies were initially considered controversial, they were proven to lower the patients’ treatment burden, thereby improving their quality of life without affecting overall survival.^15^ Without question, lumpectomies play an integral role in breast cancer patient management, but some patients are not good surgical candidates mainly because of anticipated complications from general anesthesia.

More recent advances in technology have allowed us to push the envelope of surgical de-escalation further. First, high-quality digital images detect even smaller cancers localizable by surgeons and radiologists with a short learning curve. Second, ablative therapies have made it possible to treat tumors with local anesthetics, obviating the need for general anesthesia and the surgical suite. The ablative therapies were first entertained as options for women who might not be good surgical candidates or refuse surgery.^16^ However, two large multi-institutional studies on cryoablation (one documenting tumor necrosis through ablate-resect protocol^11^ and one documenting the safety of the ablate-surveil protocol^7^) have led the way to the use of ablation as a means for further surgical de-escalation for small early-stage, low-risk breast cancers.

Because as many as half of breast cancers are diagnosed in the ageing population,^17^ surgical de-escalation is even more desirable given the higher risk of general anesthesia.^18^ The traditional approach to avoiding surgery for elderly women has been to use endocrine therapy alone because most of these tumors are ER +. However, there is evidence that the addition of local treatment of primary tumor with endocrine therapy reduces morbidity and death, offering better survival from breast cancer.^19^

The ablation of primary breast cancer provides the best of both worlds, addressing the primary tumor treatment while avoiding general anesthesia at the same time. The value of cryotherapy in addition to the use of local anesthesia alone is the natural analgesic effect of cold^20^ and enhanced anti-tumor immunity.^21^ Some techniques, such as radiofrequency ablation and laser frequency ablation, increase the temperature of the tumor and its surrounding area to induce protein denaturation and cause cell death. Cryoablation uses freeze/thaw cycles to force tumor cells to reach − 40 °C, thereby inducing cell wall necrosis and microvascular thrombosis, leading to tissue necrosis.

In this report, we describe the median age of patients as 70 years, highlighting the role of cryoablation in surgical de-escalation for the elderly population while avoiding general anesthesia but still addressing the primary tumor. Understandably, one criticism of ablative therapy is the inability to precisely document negative margins. However, cryoablation allows for a clear visualization of the edge of the ice-ball, which allows for appropriate planning of the ablation procedure. Littrup et al.^22^ specifically investigated this aspect and reported the accurate assessment of ice-ball margins beyond the tumor using MRI. We previously reported that cryoablation of early-stage, low-risk (ER +, PR +, HER2 –) unifocal invasive ductal carcinoma is safe and as effective as surgical resection, and that ice-ball margins beyond the original tumor can be reported.^6^ The next year, the 3 years interim results from the ICE3 trial of breast cancer cryoablation were published, supporting our findings.^7^

The one obvious question for proposing an office-based ablative therapy option is the role of SLNB in breast cancer. Understandably, if the patient is still subjected to a general anesthesia for the SLNB, the advantage offered by an office-based ablative procedure is minimized. Studies have shown that axillary lymph node dissection can be safely omitted in the presence of positive sentinel lymph node(s) in breast cancer patients treated with breast-conserving therapy. Because the outcome of the SLNB has no clinical consequence, the value of the procedure itself is being questioned in prospective trials.^23^ The BOOG 2013-08 is a Dutch prospective non-inferiority randomized multicenter trial investigating whether the SLNB can be safely omitted for clinically node-negative breast cancer patients.^24^ Clearly, the risk of omitting the SLNB procedure is borne by the patients at risk of having node-positive disease in the first place.

We have previously reviewed the role of SLNB given that the pre-procedure risk of node-positive disease is a function of the primary tumor size. The risk of node-positive disease is 7.8% for a clinical T1a tumor, 13.3% for a T1b tumor, and 28.5% for a T1c tumor.^25^ Hu et al.^26^ recently proposed a nomogram for predicting node-positive disease in clinical node-negative patients to potentially avoid SLNB. These authors identify high body mass index, large tumor size, high Ki-67, indistinct margins, and extensive calcifications as risk factors.

In our study, one third of the patients had tumors larger than 10 mm (cutoff at 15 mm) and well-defined ultrasound-visible masses. All were luminal A on a genomic profile with a 150-gene assay. However, we report one axillary failure at 18 months. Long-term follow-up evaluation and further studies will provide information on the safest criteria for omitting SLNB for these patients.

The de-escalation approach and a wisely chosen campaign also have investigated the option of avoiding adjuvant radiation after breast conservation. First, the long-term follow-up data from the CALGB 9343 trial further confirmed that addition of adjuvant radiation (to endocrine therapy) for women age 70 years or older with ER + early-stage breast cancer does not translate to an advantage in overall survival, distant disease-free survival, or breast preservation.^27^ Whelan et al.^28^ pushed this envelope further, including tumor biology. They hypothesized that women with small luminal A tumors could avoid radiation. The LUMINA A trial showed that women age 55 years or older with grade 1 or 2 T1N0 luminal A breast cancer after breast conservation treated with endocrine therapy alone had very low rates of local recurrence at 5 years and are candidates for omission of radiation therapy.

Second, Yang et al.^29^ analyzed a cohort of more than 5000 patients and reported that the absolute benefit of breast cancer-specific survival at 6 years attributable to adjuvant radiation is only 0.9% for patients with tumor smaller than 15 mm. Most of the patients in our study (with no tumor larger than 15 mm) as well as in the ICE3 trial were able to avoid adjuvant radiation^30^ based on clinical assessment of a multidisciplinary team. Intuitively, the zone of the ice ball in small tumors is very similar to the volume of partial breast radiation, leading physicians to be comfortable foregoing adjuvant radiation. However, only long-term follow-up evaluation will document the safety of omitting radiation for these patients, particularly those who would not have met the CALGB 9343 criteria.^31^ However, if elderly patients can avoid general anesthesia, sentinel node biopsy, and radiation therapy, it is understandable that the quality of life will be positively influenced with this ultimate de-escalation of therapy for appropriately selected patients.

It is important to note that all the patients in our series were offered adjuvant endocrine therapy. However, compliance with endocrine therapy remains an important concern and must be a part of counseling for women while de-escalation of other therapies is recommended because foregoing axillary staging and radiation of the clinically negative axilla is predicated on compliance with adjuvant endocrine therapy. The CALGB 9343 trial showed a 1% locoregional failure in the tamoxifen plus radiation arm versus 4% in the tamoxifen-alone group.^31^ Therefore, it would be prudent to believe that omitting SLNB, radiation, and endocrine therapy may be problematic and should be important in shared decision-making.

The patient perspective is an important aspect when treatment methods are examined. Many patients have anxiety surrounding their breast cancer treatment. Often, this includes undergoing surgery, the potential cost of care, and the physical changes that come with their treatment. Clinical trials have determined not only that cryoablation is just as effective as surgical resection for early-stage, low-risk tumors, but that cryoablation also provides a superior alternative when it comes to financial implications.

Cancer is one of the most expensive medical conditions to treat, and patients with cancer are more likely to experience financial burden than those with other diseases.^32,33^ The national expenditure for breast cancer in 2020 was estimated at $29.8 billion.^34^ Increased financial burden can lead to patients attempting to prioritize certain aspects of their medical care while neglecting others, based on what they can afford. Cryoablation has an 87% lower cost of care than lumpectomy ($2221.50 vs. $16,896.50).^5^ Lumpectomies conserve significantly more breast tissue than mastectomies, leading to higher breast satisfaction and psychosocial wellbeing.^35^ Cryoablation, however, provides greater physical, sexual, and cosmetic outcomes than lumpectomies, based on the BREAST-Q questionnaire.^5^ These are important and significant factors to consider in the management of early-stage, low-risk breast cancer.

As a weakness, this study was not a randomized trial. Patients meeting ablation criteria were offered cryoablation or standard-of-care resection. This non-randomization could have introduced the possibility of selection bias, by both the surgeons and the patients. This study included a small number of patients and had an average follow-up period of 15 months, which could render non-inferiority to surgery difficult to prove. However, it is important to note that 20 (61%) of the 33 cases had follow-up evaluation for at least 2 years without local failure. The ICE3 trial confirmed similar findings in a multi-institutional setting.

The strength of this study was its narrow selection criteria for cryoablation without resection. The use of ultrasound allowed precise localization of the tumor and easy visualization of the ice-ball. This study is easily replicable at a low capital cost.

As public awareness of the success of cryoablation grows, more patients when provided the option are selecting cryoablation over resection, especially unsuitable surgical candidates and the elderly who prefer to avoid surgery. However, the need for long-term follow-up evaluation remains before national guidelines embrace cryoablation as a recommended option for select patients.

## Conclusion

Earlier and increased screening has allowed breast cancer to be detected at earlier stages. This allows for increased customization of treatment and less aggressive management. De-escalation of therapy plays an important role in decreasing treatment burden and improving patient quality of life. Cryoablation eliminates the need for surgical resection for patients with early-stage, low-risk breast cancer while ensuring that no living primary tumor cells remain in situ.
